# A novel inducible haematopoietic cell‐depleting mouse model for chimeric complementation of blood cells

**DOI:** 10.1111/cpr.13472

**Published:** 2023-05-17

**Authors:** Weiyun Cao, Jiani Cao, Xing Li, Haoyu Xu, Jiayi Tian, Xiaoyan Li, Fengchao Wang, Tongbiao Zhao

**Affiliations:** ^1^ State Key Laboratory of Stem Cell and Reproductive Biology Institute for Stem Cell and Regeneration, Institute of Zoology, Chinese Academy of Sciences Beijing China; ^2^ Beijing Institute for Stem Cell and Regenerative Medicine Beijing China; ^3^ University of Chinese Academy of Sciences Beijing China; ^4^ National Institute of Biological Sciences (NIBS) Beijing China

## Abstract

Haematopoietic stem cell transplantation (HSCT) is widely used in regenerative medicine. HSCT can be used not only to treat certain types of blood cancer and immune disorders but also to induce immune tolerance in organ transplantation. However, the inadequacy of HSCs available for transplantation is still a major hurdle for clinical applications. Here, we established a novel inducible haematopoietic cell‐depleting mouse model and tested the feasibility of using chimeric complementation to regenerate HSCs and their progeny cells. Large populations of syngeneic and major histocompatibility‐mismatched haematopoietic cells were successfully regenerated by this model. The stable allogeneic chimeric mice maintained a substantial population of donor HSCs and Tregs, which indicated that the donor allogeneic HSCs successfully repopulated the recipient blood system, and the regenerated donor Tregs played essential roles in establishing immune tolerance in the allogeneic recipients. In addition, rat blood cells were detected in this model after xenotransplantation of rat whole bone marrow (BM) or Lin^−^ BM cells. This mouse model holds promise for regenerating xenogeneic blood cells, including human haematopoietic cells.

## Graphical abstract

We established a novel inducible haematopoietic cell‐depleting mouse model and tested the feasibility of using chimeric complementation to regenerate HSCs and their progeny cells. Large populations of syngeneic and major histocompatibility‐mismatched haematopoietic cells were successfully regenerated by this model. In addition, rat blood cells were detected in this model after xenotransplantation of rat whole bone marrow (BM) or Lin^−^ BM cells. This mouse model holds promise for regenerating xenogeneic blood cells, including human haematopoietic cells.

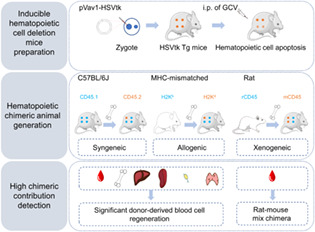

## 1 INTRODUCTION

Organ transplantation is widely used for the treatment of patients with end‐stage organ failure. However, the shortage of organs available for transplantation makes it hard to meet the existing demands,^1^ and many patients die while waiting for a transplantable organ.^2^ Although HLA matching between organs of donors and recipients is performed during transplantation, most transplanted recipients have to persevere with lifelong immunosuppressant drug regimens, which might result in a risk of life‐threatening infections.^3^, ^4^, ^5^, ^6^, ^7^, ^8^ Haematopoietic stem cell transplantation (HSCT) has been successfully used to induce tolerance during organ transplantation by the establishment of mixed haematopoietic chimerism. In addition, HSCT has been widely applied to the treatment of certain types of blood cancer and immune disorders.^9^, ^10^, ^11^, ^12^

Currently, tens of thousands of patients are cured through HSCT every year. However, the shortage of HSCs available for transplantation is still a major hurdle for this therapy. Acquiring HSCs by differentiation of pluripotent stem cells has been suggested as a promising strategy to solve this problem. Encouraging results have been obtained from HSC differentiation in vitro; however, the difficulties in functional maturation of terminally differentiated cells and low differentiation efficiency still hinder this strategy.^13^, ^14^

The Vav1 gene has been reported to be expressed in virtually all haematopoietic cell lines but in very few other cell types.^15^, ^16^, ^17^, ^18^, ^19^, ^20^ In this study, we have developed an inducible haematopoietic cell‐depleting mouse model by introducing the Vav1‐HSVtk suicide cassette into the mouse genome. This cassette expresses the herpes simplex virus (HSV) thymidine kinase (tk) gene in haematopoietic cells under control of a Vav1 regulatory element. In the presence of ganciclovir (GCV), the HSVtk protein generates a toxic derivative, which induces apoptosis in cells. By using this mouse model, we have successfully regenerated syngeneic, allogeneic, and xenogeneic blood cells by bone marrow (BM) transplantation. This strategy holds promise for regenerating xenogeneic blood cells, including human blood cells, for regenerative medicine.

## 2 MATERIALS AND METHODS

### 2.1 Animals

All animal studies were performed by the principles approved by the Institutional Animal Care and Use Committee of the Institute of Zoology, Chinese Academy of Sciences (CAS; IOZ20180060). Balb/c (H2K^d^) and C57BL/6J (CD45.2; H2K^b^) mice (6–8 weeks old) were purchased from SPF (Beijing) Biotechnology Co., Ltd. C57BL/6J (CD45.1; H2K^b^) mice were obtained from the laboratory of Liu Feng, Institute of Zoology, Chinese Academy of Sciences. C57BL/6J (CD45.1/2; H2K^b^) mice were obtained by crossing male CD45.1 and female CD45.2 mice, both on the C57BL/6J background. Male Fisher 344 (F344) rats (6–8 weeks old) were purchased from Beijing Vital River Laboratory Animal Technology Co., Ltd. Animals were housed in the standard SPF animal house of the Institute of Animal Research, Chinese Academy of Sciences.

### 2.2 Generation of HSVtk transgenic mice

The HSVtk coding sequence was amplified from pORF‐HSVtk (purchased from Beijing Huayueyang Biological) by polymerase chain reaction (PCR) and inserted into the HS21/45‐vav vector (kindly provided by Prof. Min Wang, Institute of Haematology, Chinese Academy of Medical Sciences) with SfiI and NotI restriction sites. Then, the constructed plasmid DNA was linearized by HindIII digestion and injected into pronuclei of eggs obtained from C57BL/6J‐Ly5.2 (Ly5.2, CD45.2^+^) mice. The HSVtk transgenic mice were then genotyped using PCR analysis with the primers 5′‐GAAGCCGTGAGGGCGTAACT‐3′ and 5′‐TGTTCGCGATTGTCTCGGAA‐3′.

### 2.3 Culture of spleen T cells

The spleen T cells were isolated from C57BL/6J WT or HSVtk transgenic (Tg) mice, seeded in anti‐CD3 (2 μg/mL) antibody‐coated 96‐well (1 × 10^5^ cells/well) or 6‐well (5 × 10^6^ cells/well) plates, and cultured in RPMI 1640 medium supplemented with 10% foetal bovine serum, anti‐CD28 (2 μg/mL), and IL2 cytokine (30 ng/mL). The GCV treatments (0, 20, 40, and 80 μM) were performed at day 3 of in vitro culture and continued for 72 h.

### 2.4 CCK‐8 assay

The cells were incubated with 20 μL enhanced Cell Counting Kit‐8 (CCK8) solution per well for 4 h at 37°C. Then the OD values at 450 nm and 630 nm were evaluated by a microplate reader (Bio Tek).

### 2.5 Western blot

Spleen T cells were harvested and lysed with RIPA buffer and protein concentrations were determined using a BCA assay. Equal amounts of proteins were then loaded on 12% SDS polyacrylamide gels. The separated samples were transferred to a PVDF membrane and incubated with the primary antibodies against the target proteins and then with the HRP‐conjugated secondary antibodies. The protein bands were visualised by enhanced chemiluminescence using a bioimaging analyser. The proteins were detected using antibodies against β‐actin (diluted 1:2000) and cleaved‐caspase3 (diluted 1:1000). β‐Actin was used as a loading control.

### 2.6 Flow cytometry analysis

The samples were incubated with red blood cell lysis buffer for 10 min at 4°C, then incubated with the appropriate antibodies (Table 1) at standard concentrations for 30 min. After being washed three times with phosphate‐buffered saline (PBS) and centrifuged, the cells were resuspended in 300 μL of PBS and subjected to fluorescence activated cell sorting (FACS). Data were acquired on a BD Fusion flow cytometer and analysed using FlowJo software.

**TABLE 1:** Antibodies and reagents.

Applications	Antibodies and reagents	Source	Cat #
Flow cytometry analysis	PerCP/Cyanine5.5 anti‐mouse CD3 antibody	Biolegend	100,218
	Precision count beads	Biolegend	424,902
	APC anti‐mouse/human CD45R/B220 antibody	Biolegend	103,212
	Brilliant Violet 421™ anti‐mouse/human CD11b antibody	Biolegend	101,251
	Brilliant Violet 421™ anti‐mouse Ly‐6G/Ly‐6C (Gr‐1) antibody	Biolegend	108,434
	Brilliant Violet 605™ anti‐mouse CD45.2 antibody	Biolegend	109,841
	Brilliant Violet 785™ anti‐mouse CD45.1 antibody	Biolegend	110,743
	FITC anti‐mouse Ki‐67 antibody	Biolegend	652,409
	PE/cyanine7 anti‐mouse Ly‐6A/E (Sca‐1)	Biolegend	108,113
	APC/cyanine7 anti‐mouse CD117 (c‐kit)	Biolegend	105,826
	Brilliant Violet 605™ anti‐mouse CD48	Biolegend	103,441
	Brilliant Violet 421™ anti‐mouse CD150 (SLAM)	Biolegend	115,943
	Brilliant Violet 510™ anti‐mouse CD45 antibody	Biolegend	103,137
	PE anti‐mouse H‐2K^d^ antibody	Biolegend	116,608
	True‐nuclear transcription factor buffer set	Biolegend	424,401
	APC/Fire 750 anti‐mouse CD4 antibody	Biolegend	100,459
	APC anti‐mouse CD25 antibody	Biolegend	101,909
	Alexa Fluor 488 anti‐mouse Foxp3 antibody	Biolegend	126,405
	Pacific Blue™ anti‐mouse CD45	Biolegend	103,126
	APC anti‐rat CD3 antibody	Biolegend	201,414
	APC anti‐rat CD161 antibody	Biolegend	205,606
	APC anti‐rat CD45RA Antibody	Biolegend	202,314
	APC/Cyanine7 anti‐rat CD4 Antibody	Biolegend	201,517
	PE/Cyanine7 anti‐rat CD8a Antibody	Biolegend	201,715
	PE anti‐rat CD161 antibody	Biolegend	205,604
	Pacific Blue anti‐rat CD90/mouse CD90.1 antibody	Biolegend	202,521
	Anti‐rat CD34‐PE‐Cy7	Santa Cruz	sc‐18,917 PEC7
	Anti‐rat c‐Kit‐FITC	Santa Cruz	sc‐19,619 FITC
	7‐AAD viability staining solution	Biolegend	420,403
	Mouse‐APC lineage antibody cocktail	BD	558,074
	Purified NA/LE mouse anti‐rat CD32	BD	550,273
	BV605 mouse anti‐rat CD11b/c	BD	OX‐42
	BV786 mouse anti‐rat granulocytes	BD	743,058
	BV510 mouse anti‐rat CD45	BD	740,140
	BV786 mouse anti‐rat CD45RA	BD	740,915
	Mouse/Rat SIRP alpha/CD172a APC‐conjugated Antibody	R&D	FAB7307A
	Red blood cell lysis buffer	YEASEN	40401ES76
	True‐nuclear transcription factor buffer set	Biolegend	424,401
	Annexin V‐FITC/PI apoptosis detection kit	YEASEN	40305ES50
Western blot	Caspase‐3(activated) antibody	Beyotime Biotechnology	AC033
	β‐Tubulin	Abcam	ab7291
	β‐Actin	Abcam	ab8226
	RIPA lysis buffer	Beyotime biotechnology	P0013B
	Protease inhibitor cocktail for general use, 100×	Beyotime biotechnology	P1005
	Enhanced BCA protein assay kit	Beyotime biotechnology	P0009
	0.22/0.45 μm PVDF membranes	Millipore	ISEQ00010/IPVH00010
	Enhanced chemiluminescence Western blotting substrate	Millipore	WBKLS0100
Culture of spleen T cells	Anti‐mouse CD3 SAFIRE purified	BioGems	05112–25
	Recombinant murine IL2	PeproTech	212–12
	Anti‐mouse CD28 SAFIRE purified	BioGems	10,312–25
	RPMI 1640 medium	Thermo Fisher Scientific	11,875,119
	Foetal bovine serum	Vistech	SE100‐011
	Cell counting Kit‐8 (CCK8)	Beyotime biotechnology	C0042
Neutralising antibodies	Ultra‐LEAF™ purified anti‐mouse CD4 Antibody	Biolegend	100,457
	Ultra‐LEAF™ purified anti‐mouse CD8a Antibody	Biolegend	100,763
	Ultra‐LEAF™ purified anti‐mouse NK‐1.1 antibody	Biolegend	108,759
For HSVtk Tg mice	GCV sodium	Selleck	s5065
	2 × EasyTaq® PCR SuperMix (+dye)	Transgene	AS111‐11
	QIA quick gel extraction kit	Qiagen Hilden	28,704

Apoptosis was assessed by flow cytometry. The blood cells were harvested, washed twice with PBS, and resuspended in binding buffer. An Annexin V‐FITC/PI Apoptosis Detection Kit was used according to the manufacturer's instructions. Then, cells were harvested by centrifugation at 400 g for 5 min, resuspended in 400 μL of PBS, and analysed using a BD Fusion flow cytometer.

Quantification of absolute cell number was performed by flow cytometry. Peripheral whole blood from tail bleeds was lysed by red blood cell lysis buffer for 10 min at 4°C. Precision count beads (100 μL per sample) were used to calculate the cell population of each lineage. The beads were mixed with cells in PBS for 50 μL per sample before flow cytometry analysis. Absolute cell counts per microliter were calculated based on the precision count beads.

### 2.7 Haematopoietic chimerism assay

For production of HSVtk‐BM recipient (HBR) mice, 2 × 10^6^ whole BM cells from HSVtk Tg (CD45.2) mice were injected into the tail veins of irradiated (9.0 Gy) C57BL/6J WT (CD45.1/2) recipient mice.

To generate syngeneic, allogeneic, and xenogeneic haematopoietic chimeras, the donor cells were injected into the tail veins of recipient mice, and GCV (100 mg/kg) was administered to HBR mice via intraperitoneal (i.p.) injection from day −7 to day 7 daily and every other day for the next 3 weeks.

In the construction of allogeneic and xenogeneic haematopoietic chimeras, the anti‐CD4 (200 μg per mouse), anti‐CD8 (200 μg per mouse), and anti‐NK1.1 (200 μg per mouse) neutralising antibodies were additionally administered by i.p. injection to the recipient mice 1 day before bone marrow transplantation (BMT) to help the depletion of recipient leukocytes.

### 2.8 Statistical analysis

Statistical details and number of replicates are shown in the corresponding figure legends. GraphPad Prism 7.0 (GraphPad Software Inc.) was used to perform statistical analyses. Data are expressed as mean ± standard error of the mean (SEM). Statistical significance was tested with one‐way ANOVA followed by Tukey's post hoc test, or a paired Student's *t*‐test. Statistical significance was set at *P* < 0.05. *P*‐values are indicated by asterisks as follows: *P < 0.05, **P < 0.01, ***P < 0.001, and ****P < 0.0001.

## 3 RESULTS

### 3.1 Generation of a mouse model with inducible depletion of haematopoietic cells

**FIGURE 1:**
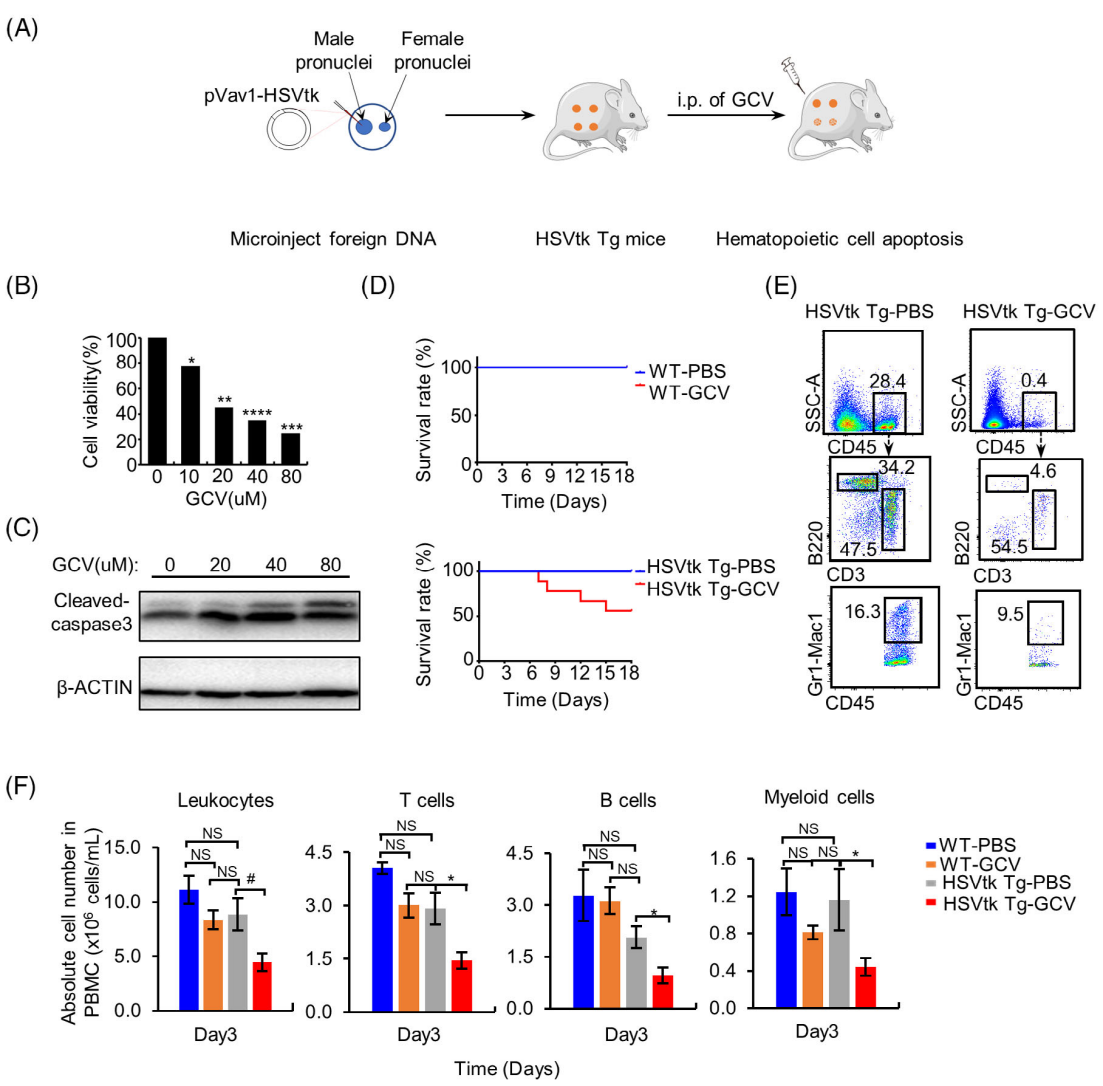
Generation and identification of HSVtk transgenic (Tg) mice. (A) Schematic of the procedure for constructing the HSVtk Tg mice and depleting the blood cells. The model is constructed by introducing the Vav1‐HSVtk suicide cassette into the murine genome. In the resulting Tg mice, the haematopoietic cells can be induced to undergo apoptosis by injection of ganciclovir (GCV). (B) In spleen cells isolated from HSVtk Tg mice, apoptosis was induced by GCV treatment in a dose‐dependent manner. (C) Western blot analyses indicate that the level of the apoptotic marker protein cleaved caspase3 in HSVtk Tg spleen T cells is up‐regulated by increasing doses of GCV. The protein expression level is normalised to that of β‐Actin. (D) Survival rates of Phosphate‐buffered saline (PBS)‐ or GCV‐treated mice. (E,F) fluorescence activated cell sorting analysis of the absolute numbers of leukocytes (CD45^+^), T cells (CD3^+^), B cells (B220^+^), and myeloid cells (Gr1‐Mac1^+^) in peripheral blood mononuclear cells of each group at day 3 of GCV or PBS treatment. NS, not significant; WT‐PBS, wild‐type mice treated with PBS; HSVtk Tg‐PBS, HSVtk Tg mice treated with PBS; WT‐GCV, wild‐type mice treated with GCV at a dose of 100 mg/kg; HSVtk Tg‐GCV, HSVtk Tg mice treated with GCV at a dose of 100 mg/kg. *n* = 9 mice per group, data are shown as the mean values ± SEM. *, *P* < 0.05; **, *P* < 0.01; ***, *P* < 0.001; ****, *P* < 0.0001, #, *P* = 0.054 (on the cusp of conventional statistical significance).

To test the feasibility of regenerating blood cells using chimeric complementation, we generated a transgenic (Tg) mouse model with inducible depletion of the haematological system, in which the expression of the suicide gene HSVtk is driven by the murine Vav1 gene regulatory element HS21/45. In this model, the haematopoietic cells can be induced into apoptosis by injection of GCV (Figure 1A). As expected, the spleen cells isolated from this mouse were killed by GCV treatments in a dose‐dependent manner (Figure 1B). Accordingly, the level of cleaved caspase3 protein in HSVtk Tg spleen T cells was up‐regulated by increasing doses of GCV (Figure 1C).

Next, we performed a titration assay to determine the safe dosages for GCV treatment in vivo (Figures S1 and S2). Wild‐type (WT) mice were continuously treated with GCV at dosages of 0, 20, 40, 80, or 160 mg/kg for 15 days. Cell apoptosis and mouse body weight were measured every 3 days during the titration period, and tissue histology was evaluated at the end of the titration assay (Figure S1A). The results showed that there were no significant differences in cell apoptosis between the GCV treatment groups and the control groups at the tested dosages (Figure S1B); however, the body weight of the 160 mg/kg group was significantly reduced compared with the control group (Figure S1C). The histological data indicated that the cortex and medulla of the thymus tissue were dramatically damaged in the 160 mg/kg group (Figure S1D). These results indicated that the maximum safe dosage for GCV administration was likely between 80 and 160 mg/kg. Then we performed a safety evaluation on the treatment dosage of 100 mg/kg GCV (Figure S2A). The results showed that 15 days of continuous treatment with 100 mg/kg GCV did not induce a significant decrease in cell viability, mouse body weight, or tissue weight compared with the control group (Figures S2B–S2D). No obvious damage was observed in the histological structure of the liver, kidney, thymus, or spleen in the GCV‐treated mice (Figure S2E). Therefore, the dose of 100 mg/kg of GCV was used in the following in vivo experiments.

Next, the HSVtk Tg mice and WT mice were continuously treated with PBS or GCV on days 1–15, then sacrificed on day 18. The PBS‐treated HSVtk Tg mice, GCV‐treated WT mice, and PBS‐treated WT mice showed 100% survival rates until day 18, while only half of the GCV‐treated HSVtk Tg mice survived (Figure 1D). The absolute numbers of leukocytes including T cells, B cells, and myeloid cells in peripheral blood mononuclear cells (PBMCs) of the GCV‐treated HSVtk Tg group were significantly lower than that of PBS‐treated HSVtk Tg group and the GCV/PBS‐treated WT groups (Figure 1E,F). These data indicated that the blood cells in HSVtk Tg mice were effectively depleted by GCV treatment.

The male HSVtk Tg mice exhibited a sterile phenotype, which is consistent with the previous findings that HSV infection and the accumulation of HSVtk in the mouse body can induce male sterility.^21^, ^22^, ^23^ This breeding deficiency severely decreased the yield of HSVtk Tg mice. To compensate for the inadequacy, we transplanted BM from HSVtk Tg mice (CD45.2) into myeloablative WT C57BL/6J (CD45.1/2) mice for further experiments (Figure S3A). At 4 weeks after BMT, we used FACs to measure the chimeric proportions, that is, the percentage of donor cells, in peripheral blood of recipient mice. The chimeric proportions of T cells, B cells, and myeloid cells in the peripheral blood of recipients were 40.9% ± 0.7% (CD45.2^+^ CD3^+^), 98.4% ± 0.1% (CD45.2^+^ B220^+^), and 90.7% ± 0.8% (CD45.2^+^ CD11b^+^ Gr1/Mac1^+^), respectively (Figures S3B and S3C). The BM cells from one HSVtk Tg mouse were able to generate 10 HSVtk‐BM recipient (HBR) mice (designated HBR mice), which were used in the subsequent blood chimerism assays.

### 3.2 GCV‐induced haematopoietic cell depletion can be successfully compensated by syngeneic BMT

**FIGURE 2:**
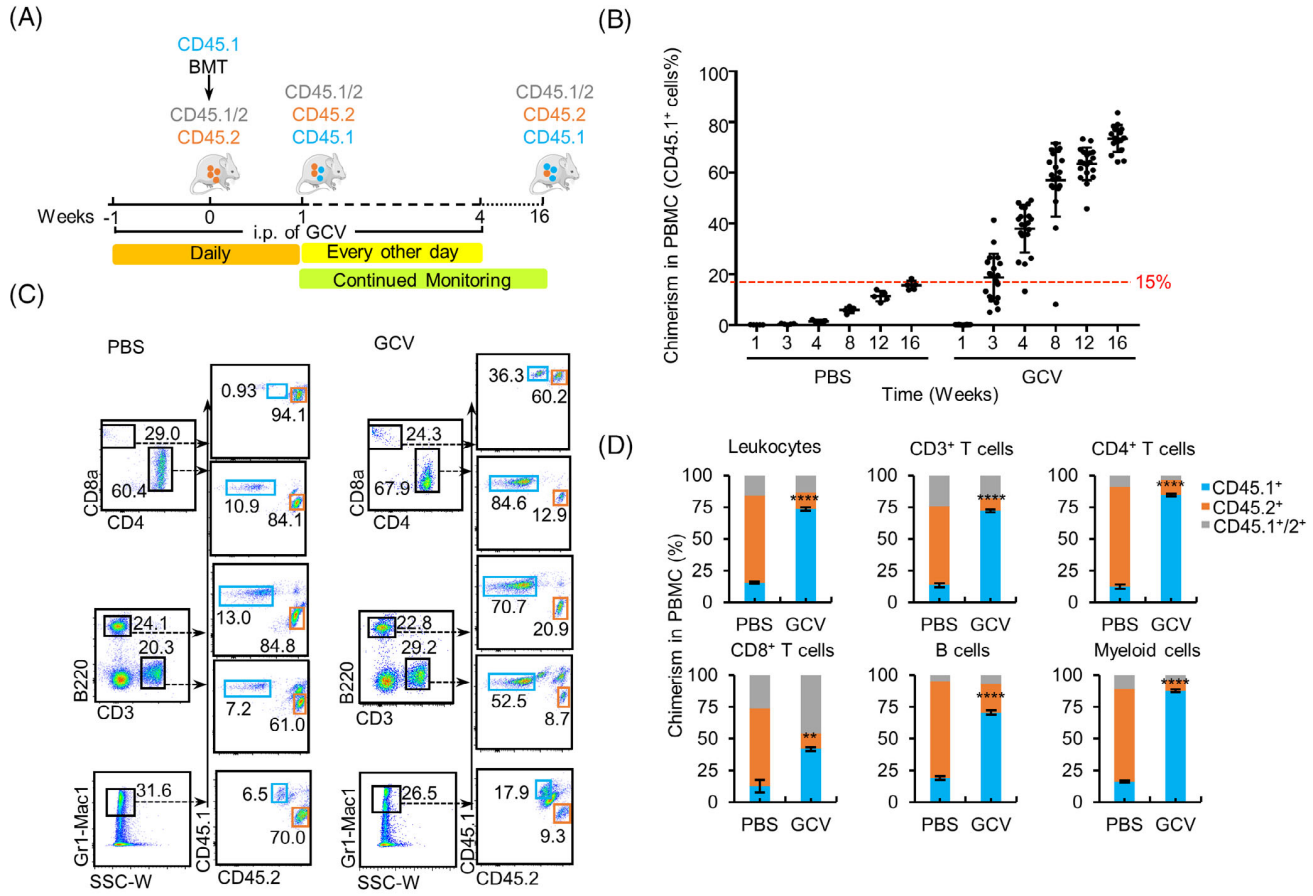
Generation of syngeneic haematopoietic chimeras. (A) Schematic of the procedure for constructing the syngeneic haematopoietic chimeras; 1.5 × 10^6^ C57BL/6J (CD45.1) bone marrow cells were transplanted into HSVtk‐BM recipient (HBR) mice (CD45.2) at day 0, and intraperitoneal injection of ganciclovir (GCV) was performed daily from week −1 to week 1 and every other day during weeks 2–4. Fluorescence activated cell sorting (FACS) analyses were performed periodically to monitor the level of chimerism in peripheral blood mononuclear cells (PBMCs). (B) Summary of the haematopoietic chimerism in mice from the GCV group and the phosphate‐buffered saline (PBS) group at each time point after bone marrow transplantation (BMT). Each dot represents the percentage of CD45.1^+^ cells in the PBMCs of an individual mouse. (C) FACS analysis of the percentage of chimeric T cells (CD3^+^, CD4^+^, or CD8^+^), B cells (B220^+^), and myeloid cells (Gr1‐Mac1^+^) in the PBMCs of mice from the GCV and PBS groups at the 16th week after BMT. (D) Statistical analysis of the level of chimerism (percentage of CD45.1^+^) of CD45^+^ leukocytes, CD3^+^ T cells, CD4^+^ T cells, CD8^+^ T cells, and Gr1‐Mac1^+^ myeloid cells in the GCV and PBS groups at the 16th week after BMT (GCV, *n*_initial_ = 20, *n*_surviving at week 16_ = 16; PBS, *n* = 5; data are shown as the mean values ± SEM, **,*P* < 0.01; ***,*P* < 0.001; ****, *P* < 0.0001).

To test the feasibility of using HBR mice for chimeric complementation of blood cells, syngeneic BM cells from C57BL/6J mice (CD45.1) were first transplanted into GCV‐treated HBR mice (CD45.2), and the chimeric proportion of CD45.1 cells in the PBMCs of recipient animals was analysed periodically (Figure 2A,B). In the GCV‐treated mice, chimeric CD45.1 haematopoietic cells ranging from 5.0% to 41.4% (18.8% ± 2.1%) were detected at the end of the third week after BMT. The chimeric proportions increased to 73.5% ± 1.3% at the end of 16 weeks after transplantation. In the control group, a very limited level of CD45.1 chimerism (5.9% ± 0.5%) was detected until the eighth week post‐transplantation, and the level increased to 15.6% ± 0.7% at the end of the 16th week after BMT, which is significantly lower than the GCV‐treated group (Figure 2B). The chimeric proportions of each CD45.1 haematopoietic lineage were analysed at the end of the 16th week after BMT. In contrast to the PBS control groups, which showed limited CD45.1 chimerism, the GCV‐treated mice showed significantly higher CD45.1 chimerism of leukocytes including T cells, B cells, and myeloid cells (Figure 2C,D). Similar results were observed in the BM, thymus, lymph nodes, liver, and spleen of recipient mice (Figure S4A). The chimeric ratio of CD45.1 regulatory T cells (Tregs) reached 80.5% ± 3.3% in the thymus tissues of the GCV group, significantly higher than that of the PBS group (Figure S4B). 80% of the mice in the GCV group survived at the 16th week after BMT, while the survival rate of the PBS group was 100% (Figure S4C). Together, these data indicated that the transplanted CD45.1 haematopoietic cells, most likely including HSCs, are able to survive and repopulate the BM of recipient HBR mice.

### 3.3 Allogeneic haematopoietic chimeras exhibit a high substitution level of donor HSCs and Tregs

**FIGURE 3:**
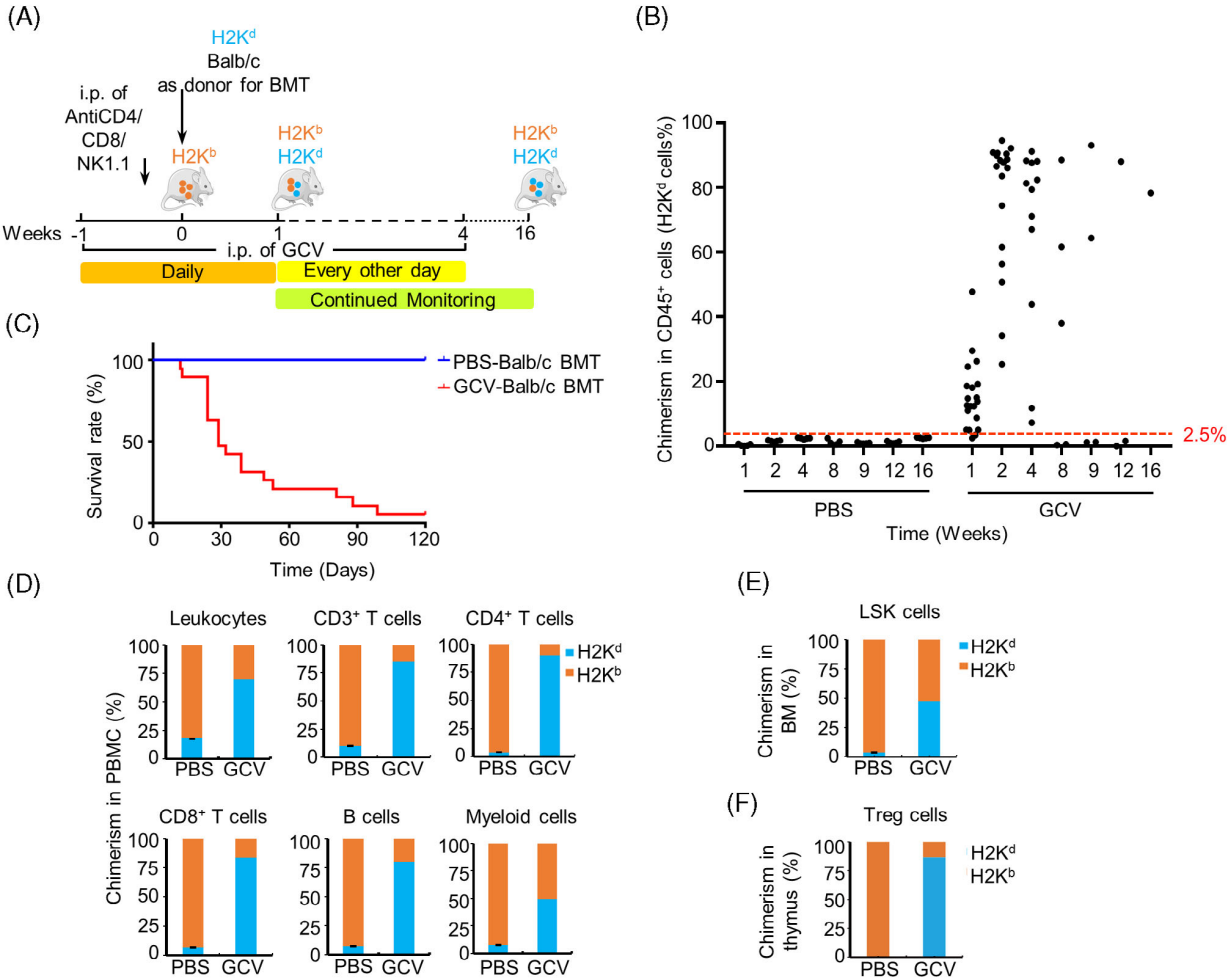
Generation of major histocompatibility (MHC)‐mismatched haematopoietic chimeras. (A) Schematic of the procedure for constructing the allogeneic (MHC‐mismatched) haematopoietic chimeras; 10 × 10^6^ Balb/c (H2K^d^) bone marrow cells were transplanted into HSVtk‐BM recipient mice (H2K^b^) at day 0. Neutralising antibodies (anti‐CD8/CD4/NK1.1) were administered by intraperitoneal (i.p.) injection 1 day before bone marrow transplantation (BMT), and i.p. injection of ganciclovir (GCV) was performed daily from week −1 to week 1 and every other day during week 2 to week 4. Fluorescence activated cell sorting (FACS) analyses were performed periodically to monitor the level of chimerism in peripheral blood mononuclear cells (PBMCs). (B) Summary of the level of haematopoietic chimerism in mice from the GCV group and the phosphate‐buffered saline (PBS) group at each time point after BMT. Each dot represents the percentage of H2K^d^ cells in the PBMCs of an individual mouse. (C) Survival curves of the mice in the GCV and PBS groups after BMT. (D) Chimerism levels (percentage of H2K^d^ cells) in CD45^+^ leukocytes, B220^+^ B cells, Gr1‐Mac1^+^ myeloid cells, CD3^+^ T cells, CD4^+^ helper T cells, and CD8^+^ cytotoxic T cells in PBMCs of GCV‐ or PBS‐treated mice. FACS analysis was carried out at the 16th week after BMT. (E) Chimerism levels (percentage of H2K^d^ cells) of Lineage^−^ Sca1^+^ c‐Kit^+^ cells (LSK) in the BM of GCV‐ or PBS‐treated mice. FACS analysis was performed at 16 weeks after BMT. (F) Chimerism levels (percentage of H2K^d^ cells) of CD4^+^ CD25^+^ FOXP3^+^ Treg cells in the thymus tissues of GCV‐ or PBS‐treated mice. FACS analysis was carried out at 16 weeks after BMT. (GCV, *n*_initial_ = 20, *n*_surviving at week 16_ = 1; PBS, *n* = 5; data are shown as the mean values ± SEM).

We next tested whether allogeneic haematopoietic cells can repopulate the recipients by transplanting BM cells of Balb/c mice (H2K^d^) into GCV‐treated HBR mice (H2K^b^) (Figure 3A). Anti‐CD4, anti‐CD8, and anti‐NK1.1 neutralising antibodies were additionally injected into the recipient mice one day before BMT to enhance the depletion of recipient leukocytes (Figure S5). The results showed that transplanted H2K^d^ haematopoietic cells accounted for 15.3% ± 2.3% (ranging from 2.5% to 47.8%) of total CD45^+^ cells in the PBMCs of recipient mice at the end of the first week after BMT. The H2K^d^ chimeric proportion increased to higher than 80% in 12 of 18 transplanted HBR mice at the end of the second week after BMT, with the maximum chimeric proportion at 94.5% (Figure 3B). Four weeks after transplantation, severe immune rejection responses were observed in recipient mice, resulting in high mortality rates (Figure 3C). At the end of the 16th week after BMT, there was only one surviving mouse in the GCV‐treated group, with a 70.2% H2K^d^ chimeric proportion in PBMCs (Figure 3B,C).

At the end of the 16th week after BMT, the H2K^d^ chimerism proportions of each haematopoietic lineage in PBMCs, BM, thymus, lymph nodes, liver, and spleen were analysed by a FACS. In contrast to the PBS control group, which showed limited H2K^d^ chimerism, the single surviving GCV‐treated recipient mouse exhibited high H2K^d^ chimerism of CD45^+^ leukocytes, CD3^+^ T cells (70.2%), CD4^+^ T cells (65.3%), CD8^+^ T cells (92.0%), B220^+^ B cells (84.4%), and myeloid cells (82.0%) in PBMCs (Figures 3D and S6A). Similar results were observed in the BM, thymus, lymph nodes, liver, and spleen of the recipient mouse (Figure S6D).

Haematopoietic stem cells (HSCs) hold the ability to reconstitute all blood cell types. HSCs are enriched in the Lin^−^Sca1^+^c‐kit^+^ (LSK) cell population in mouse BM.^24^ The proportion of donor‐derived H2K^d^ LSK cells was 46.9% in the GCV‐treated HBR recipient mouse, whereas the H2K^d^ LSK chimerism in the PBS control group was 3.6% ± 0.4% at the end of the 16th week after BMT (Figures S6B and 3E). These data suggest that the donor‐derived HSCs integrated into the recipient haematopoietic niche and participated in haematopoietic reconstitution in the HBR recipient mice.

Tregs play important roles in immune tolerance. Interestingly, the donor‐derived H2K^d^ Tregs (CD4^+^ CD25^+^ Foxp3^+^) account for 86.6% of all Tregs in the thymus of GCV‐treated HBR recipient mice (Figures 3F and S6C). These data indicate that donor‐derived Tregs might provide a sufficiently tolerant environment for the survival and repopulation of transplanted H2K^d^ BM cells.

### 3.4 Regeneration of rat haematopoietic cells in HBR mice

**FIGURE 4:**
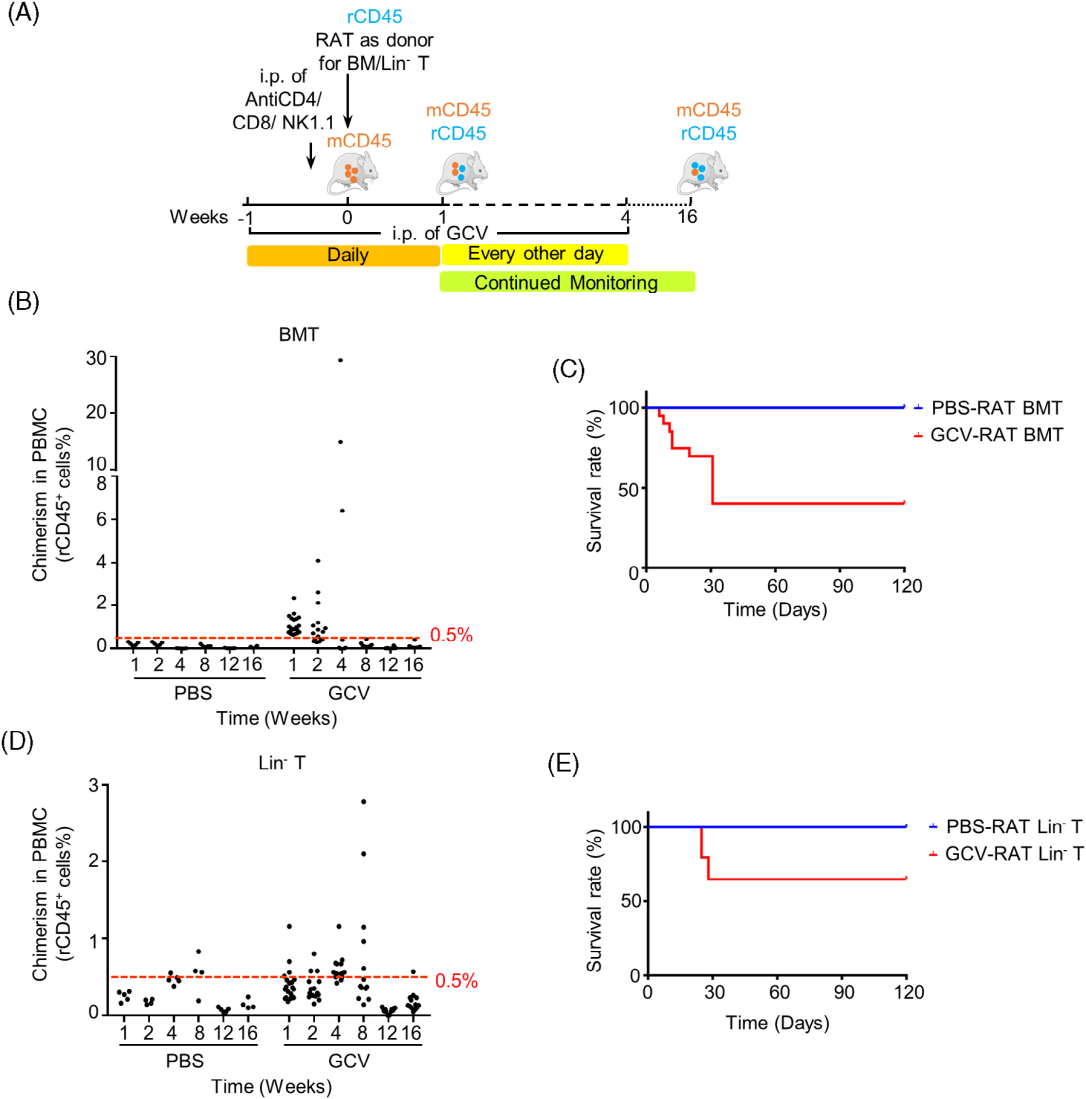
Xenogeneic haematopoietic chimeras are obtained under the HSVtk/ganciclovir (GCV) system after the transplantation of rat bone marrow cells into HSVtk‐BM recipient (HBR) mice. (A) Schematic of the procedure for constructing the xenogeneic (rat‐mouse) haematopoietic chimeras; 15 × 10^6^ rat (rCD45) whole bone marrow cells or 8 × 10^5^ rat (rCD45) Lineage^−^ cells (Lin^−^: rCD45^+^ rCD3^−^ rCD45RA^−^ rCD161^−^ rSIRPA^−^) were transplanted into HBR mice (mCD45) at day 0. Neutralising antibodies (anti‐CD8/CD4/NK1.1) were administered via intraperitoneal (i.p.) injection 1 day before BMT, and i.p. injection of GCV was performed daily from week −1 to week 1 and every other day from week 2 to week 4. Fluorescence activated cell sorting analyses were performed periodically to monitor the level of chimerism in peripheral blood mononuclear cells (PBMCs). (B,D) Summary of haematopoietic chimerism (percentage of rCD45^+^ cells in PBMCs) in mice from the GCV group and the PBS group at each time point after transplantation of rat bone marrow cells (B: BMT) or rat Lin^−^ cells (D: Lin^−^ T). (C,E) Survival curves of the mice from the GCV group and the PBS group at each time point after transplantation of rat bone marrow cells (C: BMT) or rat Lin^−^ cells (E: Lin^−^ T). (BMT: GCV, *n*_initial_ = 20, *n*_surviving at week 16_ = 8; PBS, *n* = 5; Lin^−^ T: GCV, *n*_initial_ = 20, *n*_surviving at week 16_ = 13).

To test the feasibility of using HBR mice to regenerate rat haematopoietic cells, we transplanted BM cells of inbred Fischer (F344) rats into GCV‐treated HBR recipient mice (Figure 4A). Anti‐CD4, anti‐CD8, and anti‐NK1.1 neutralising antibodies were additionally injected into the recipient mice one day before BMT to enhance the engraftment of rat haematopoietic cells. Encouragingly, rat haematopoietic cells implanted in GCV‐treated HBR mice accounted for 1.1% ± 0.1% (ranging from 0.7% to 2.4%) of mouse and rat CD45^+^ (mrCD45^+^) cells in PBMCs at the end of the first week after BMT. The percentage of mrCD45^+^ cells rose as high as 29.4% at the end of the 4th week after transplantation. Around half of HBR recipient mice died 4 weeks after BMT, and the percentage of implanted haematopoietic cells in the PBMCs of HBR mice gradually declined along with GCV withdrawal in the surviving HBR recipient mice (Figures 4B,C and S6E).

We next used lineage^−^ (Lin^−^) BM cells of F344 rats, which might potentially be enriched in rat HSC populations, as donor cells for this xenotransplantation. The maximum chimeric proportion of rCD45^+^ cells in PBMCs of GCV‐treated HBR recipients reached 1.2% at the end of the first week after BMT and 2.8% at the end of the eighth week after transplantation. The rat haematopoietic cells in PBMCs of GCV‐treated HBR mice declined to 0.5% in the 12th week after transplantation (Figures 4D and S6E). 65% of transplanted recipient mice survived through 16 weeks after Lin^−^ BMT, with <0.5% of chimeric rat haematopoietic cells (Figures 4D,E and S6E).

Together, these data indicate that HBR mice can be used to regenerate xenogeneic rat haematopoietic cells, although with low levels of chimerism.

## 4 DISCUSSION

The HSVtk/GCV suicide system has been widely used in cancer gene therapy.^25^, ^26^, ^27^, ^28^, ^29^, ^30^ In this system, thymidine kinase phosphorylates intravenously administered GCV into a toxic compound that is incorporated into replicating DNA by DNA polymerase, causing termination of synthesis and the selective killing of dividing cells by activation of apoptosis pathways. In this study, we generated a mouse model (HBR) with inducible haematological depletion in which the expression of the suicide gene HSVtk is controlled by the regulatory element HS21/45 of Vav1. The haematopoietic cells in HBR mice can be specifically depleted by GCV administration (Figure 1). Using this model, we successfully generated syngeneic, allogeneic, and xenogeneic haematopoietic chimeric mice. The detection of significant levels of donor‐derived LSK cells and haematopoietic lineage cells in BM, peripheral blood, thymus, lymph nodes, liver, and spleen of syngeneic and allogeneic recipient HBR mice indicates that the donor HSCs can successfully repopulate and reconstitute the recipient blood system (Figures 2C,D, 3D,E, S4A, and S6).

Aplastic anaemia is a disease of BM failure, and the clinical diagnosis is based on pancytopenia.^31^ Previous studies showed that aplastic anaemia mice without therapeutic treatment suffered a 100% mortality rate within 45 days.^32^, ^33^ Similarly, the GCV‐treated HSVtk Tg mice exhibited a significant decline in absolute number of leukocytes in PBMCs (Figure 1E,F), and only 50% of HSVtk Tg mice survived 18 days after GCV treatment (Figure 1D). While after transplantation of syngeneic BM, significant donor reconstitution in the haematopoietic lineages (CD45.1^+^ T cells, B cells, and myeloid cells) can be detected in BM, thymus, lymph nodes, liver, and spleen of GCV‐treated recipient mice (Figure 2C,D and S4A) 16 weeks after BMT. In addition, the GCV‐treated syngeneic BMT recipient mice maintained 100% survival rate for 58 days and remained a survival rate above 80% over 16 weeks after BMT (Figure S4C), indicating syngeneic BMT can rescue the effects of blood cell clearance caused by GCV treatment.

Immune rejection is a major challenge during the construction of haematopoietic chimeras. In the allogeneic and xenogeneic chimeric assays, the survival rates of transplanted HBR mice were significantly lower than that of control groups, and several of the surviving mice had lost the donor haematopoietic cells at the late stages of the experiment, judged by periodical analysis of peripheral blood (Figures 3 and 4). Tregs play a key role in immune tolerance during organ transplantation and infusion of donor Tregs to the recipient has been used as a strategy to alleviate transplant rejection.^34^, ^35^, ^36^, ^37^ There are several different sources of Tregs, among which thymus‐derived Tregs (nTregs) have a better immunosuppressive effect, especially in the formation of peripheral tolerance.^38^ In the allogeneic experiment, high chimerism of donor‐derived Tregs was detected in the thymus tissues of recipient mice at 16 weeks after BMT (Figures S4B and 3F). These results are consistent with a high chimeric ratio of cells from differentiated haematopoietic lineages in HBR PBMCs (Figures 2D and 3D). Together, these data support the notion that donor derived Tregs play critical roles in tolerance induction.

In this study, we successfully obtained rat‐mouse xenogeneic haematopoietic chimeras using HBR mice. The maximum proportion of haematopoietic chimerism reached 29.4% (Figure 4B). The rat haematopoietic cells were maintained for 4 weeks in HBR mice when transplanting whole BM, and for 8 weeks when transplanting Lin^−^ BM cells of F344 rats (Figure 4B,D). These encouraging data suggest that this model can be expanded to human haematopoietic cells.

In conclusion, we have successfully developed an inducible haematopoietic cell‐depleting mouse model, which can be used for the regeneration of allogeneic and xenogeneic haematopoietic cells. Although a relatively high ratio of allogeneic haematopoietic chimerism was achieved, the xenografted rat haematopoietic cells showed a limited chimerism ratio. Further optimization of suicide induction conditions including the drug dosage, treatment time, and combination of other immunosuppressive methods might help to improve the chimerism of xenografts.

## AUTHOR CONTRIBUTIONS

*Designed the project*: Tongbiao Zhao, Weiyun Cao, and Jiani Cao. *Performed the experiments*: Weiyun Cao, Xing Li, Haoyu Xu, and Jiayi Tian. *Collected research data and did statistical analysis*: Weiyun Cao. *Supervised the experiments*: Tongbiao Zhao. *Wrote and edited the manuscript*: Weiyun Cao, Jiani Cao, and Tongbiao Zhao. All authors reviewed the manuscript.

## FUNDING INFORMATION

This work was supported by grants from the Strategic Priority Research Program of the Chinese Academy of Sciences (XDA16030302 and XDA16040501), the National Key R&D Program of China (2022YFA1103601 and 2018YFA0108402), the Strategic Collaborative Research Program of the Ferring Institute of Reproductive Medicine (Grant No. 33), and the National Natural Science Foundation of China Program (31720103907, 31570995, and 31621004). The authors thank Min Wang Lab from the Institute of Haematology, Chinese Academy of Medical Sciences, for donating the HS21/45‐vav1 backbone vector.

## CONFLICT OF INTEREST STATEMENT

The authors declare no competing financial interests.

## Supporting information


**Figure S1. Titration to determine the safe dose for GCV treatment of mice in vivo.** (A) Schematic of the GCV toxicity test in WT C57BL/6J mice. WT C57BL/6J mice were intraperitoneally injected with different doses of GCV (20 mg/kg, 40 mg/kg, 80 mg/kg, and 160 mg/kg) or PBS daily for 15 days. The viability of BM cells and PBMCs was measured on day 0, day 3, day 6, day 9, day 12, and day 15 by staining with annexin V and PI. The body weights were measured every 3 days. Haematoxylin and eosin (H&E) staining of tissue sections from each group of mice was performed on the 15th day. (B) There is no significant difference in the level of cell apoptosis between the GCV treatment groups and the control group. (C) The body weight of mice in the 160 mg/kg group is significantly reduced compared with the control group. (D) The thymic cortex and medulla are severely damaged in the 160 mg/kg group. Scale bar: 200 μm. (n = 20; data are shown as the mean values±SEM; *, P < 0.05; **, P < 0.01; ****P < 0.0001)

**Figure S2. Safety evaluation of the GCV treatment dose of 100 mg/kg.** (A) Schematic illustration of the experimental design to test the toxicity of administering 100 mg/kg GCV for 15 days. WT C57BL/6J mice were treated with GCV (100 mg/kg) or PBS once a day for 15 days by peritoneal injection. The viability of BM cells and PBMCs was analysed on day 0, day 3, day 6, day 9, day 12, and day 15 by staining with annexin V and PI. The body weights were measured every 3 days. Tissue weights were measured on day 0, day 3, day 6, day 9, day 12, and day 15. H&E staining of tissue sections from each group of mice was performed on the 15th day. (B‐E) 15 days of continuous 100 mg/kg GCV treatment does not induce a significant decrease in cell viability (B), body weight (C), tissue weight (D), or tissue structure (E) compared with the control group. Scale bar: 200 μm. Data are shown as the mean values±SEM, n = 20; *, P < 0.05; **, P < 0.01; ***, P < 0.001; ****, P < 0.0001; ns, not significant.

**Figure S3. HBR mice are generated by transplantation of bone marrow cells into HSVtk Tg mice.** (A) Schematic of the procedure for constructing HBR mice. (B) Schematic overview of the FACS gating strategy. The left panels show the FACS sorting of PBMCs: B220^+^ B cells, Gr1‐Mac1^+^ myeloid cells, and CD3^+^ T cells. These populations are then gated for CD45.1^+^ cells, CD45.2^+^ cells, and CD45.1^+^/2^+^ cells (right panels). (C) The percentage of CD45.2^+^ cells and CD45.1^+^/2^+^ cells in HSVtk‐BM recipient (HBR) mice, determined by FACS as shown in B. T: T cells, marker is CD3^+^; B: B cells, marker is B220^+^; M: myeloid cells, marker is Gr1‐Mac1^+^. Data are shown as the mean values±SEM, n = 133.

**Figure S4. Mice receiving syngeneic transplants exhibit a high level of donor cell chimerism and a high survival rate.** (A) T cells (CD3^+^/CD4^+^/CD8^+^ cells), B cells (B220^+^ cells), and myeloid cells (Gr1‐Mac1^+^ cells) in different tissues from the recipient mice in the GCV and PBS groups at 16 weeks after the transplantation of bone marrow from C57BL/6J (CD45.1) donor mice. (B) Statistical analysis of the chimerism ratios (CD45.1^+^ cell %) of CD4^+^ CD25^+^ FOXP3^+^ Treg cells in the thymus tissues detected by FACS analysis at the 16th week after BMT. (C) Survival curves of the mice in GCV and PBS group after syngeneic BMT. (GCV, n_initial_ = 20, n_surviving at week 16_ = 16; PBS, n = 5; data are shown as the mean values±SEM; **,P < 0.01; ****, P < 0.0001)

**Figure S5. CD4** ^**+**^ **T cells, CD8** ^**+**^ **T cells, and NK cells in the recipient mice are blocked by intraperitoneal injection of neutralising antibodies.** (A) Schematic of the procedure for titrating the neutralising antibodies. WT C57BL/6J mice were intraperitoneally injected with neutralising antibodies (anti‐CD4: 200 μg; anti‐CD8: 200 μg; and anti‐NK1.1: 200 μg) or PBS at day 0. The percentage of CD4^+^ helper T cells, CD8^+^ cytotoxic T cells, and CD49b^+^ natural killer cells (NK cells) in the PBMCs were analysed on day 7 (control group: PBS; experimental group: Antibodies). (B) The distributions of CD4^+^ T cells, CD8^+^ T cells, and CD49b^+^ NK cells in the PBMCs from each group of mice were detected by FACS at day 7 after treatment. (C) The percentages of CD4^+^ T cells, CD8^+^ T cells, and NK cells are significantly lower in the Antibodies group than in the PBS group. (n = 5, mean values±SEM, ****P < 0.0001).

**Figure S6. Mice receiving allogeneic transplants exhibit a high level of donor cell chimerism in different tissues.** (A) FACS analysis of the chimerism of T cells (CD3^+^/CD4^+^/CD8^+^ cells), B cells (B220^+^ cells), and myeloid cells (Gr1‐Mac1^+^ cells) in PBMCs from the recipient mice in the GCV and PBS groups at 16 weeks after transplantation of bone marrow (BM) cells from Balb/c (H2K^d^) donor mice. (B) Representative FACS diagram of chimerism of LSK cells (Lineage^−^ Sca1^+^ c‐Kit^+^) in BM of the GCV and PBS groups at 16 weeks after the transplantation of BM from Balb/c (H2K^d^) donor mice. (C) FACS analyses of Treg cells (CD4^+^ CD25^+^ Foxp3^+^) in thymus tissues from the mice in the GCV and PBS groups at 16 weeks after receiving BM transplants from Balb/c (H2K^d^) donors. (D) Chimerism levels of T cells (CD3^+^/CD4^+^/CD8^+^ cells), B cells (B220^+^ cells), and myeloid cells (Gr1‐Mac1^+^ cells) in different tissues from the mice in the GCV and PBS groups at 16 weeks after receiving BM transplants from Balb/c (H2K^d^) donors. (E) Representative FACS diagram of the chimerism level of rat CD45 (rCD45) cells in PBMCs from each group of mice at 16 weeks after transplantation of BM or Lin^−^ cells from F344 rats. (GCV, n_initial_ = 20, n_surviving at week 16_ = 1; PBS, n = 5; data are shown as the mean values±SEM).

## Data Availability

The raw data supporting the conclusions of this article will be made available by the authors, without undue reservation.
