# Supplementary figures and images for: A novel inducible haematopoietic cell‐depleting mouse model for chimeric complementation of blood cells
Source: Cell Prolif. 2023 May 17;56(5):e13472. doi: 10.1111/cpr.13472 (PMC10212702; doi:10.1111/cpr.13472)

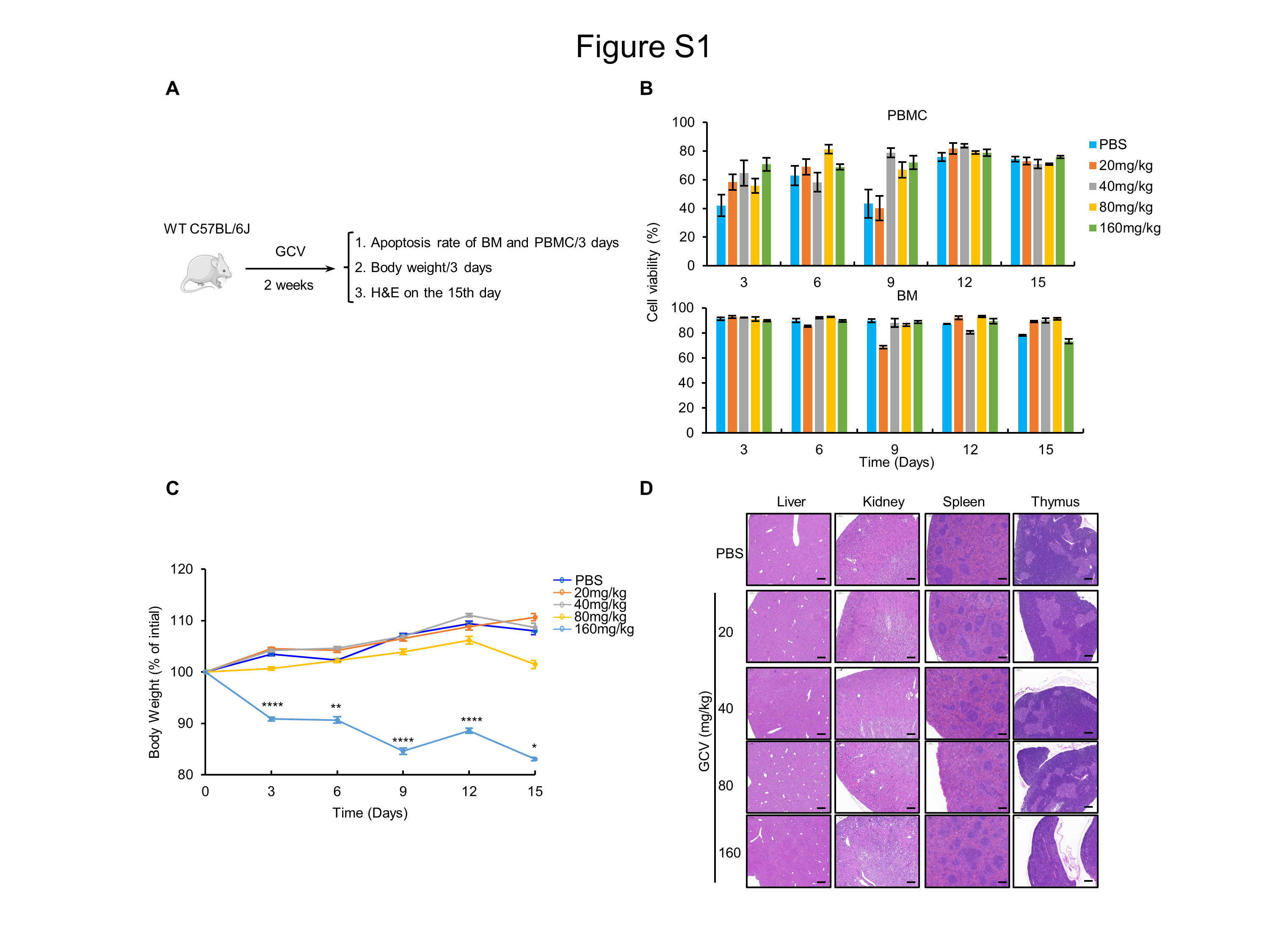

Supplement: Supplementary file 1 — Figure S1. Titration to determine the safe dose for GCV treatment of mice in vivo. (A) Schematic of the GCV toxicity test in WT C57BL/6J mice. WT C57BL/6J mice were intraperitoneally injected with different doses of GCV (20 mg/kg, 40 mg/kg, 80 mg/kg, and 160 mg/kg) or PBS daily for 15 days. The viability of BM cells and PBMCs was measured on day 0, day 3, day 6, day 9, day 12, and day 15 by staining with annexin V and PI. The body weights were measured every 3 days. Haematoxylin and eosin (H&E) staining of tissue sections from each group of mice was performed on the 15th day. (B) There is no significant difference in the level of cell apoptosis between the GCV treatment groups and the control group. (C) The body weight of mice in the 160 mg/kg group is significantly reduced compared with the control group. (D) The thymic cortex and medulla are severely damaged in the 160 mg/kg group. Scale bar: 200 μm. (n = 20; data are shown as the mean values±SEM; *, P < 0.05; **, P < 0.01; ****P < 0.0001) [file CPR-56-e13472-s001.tif]

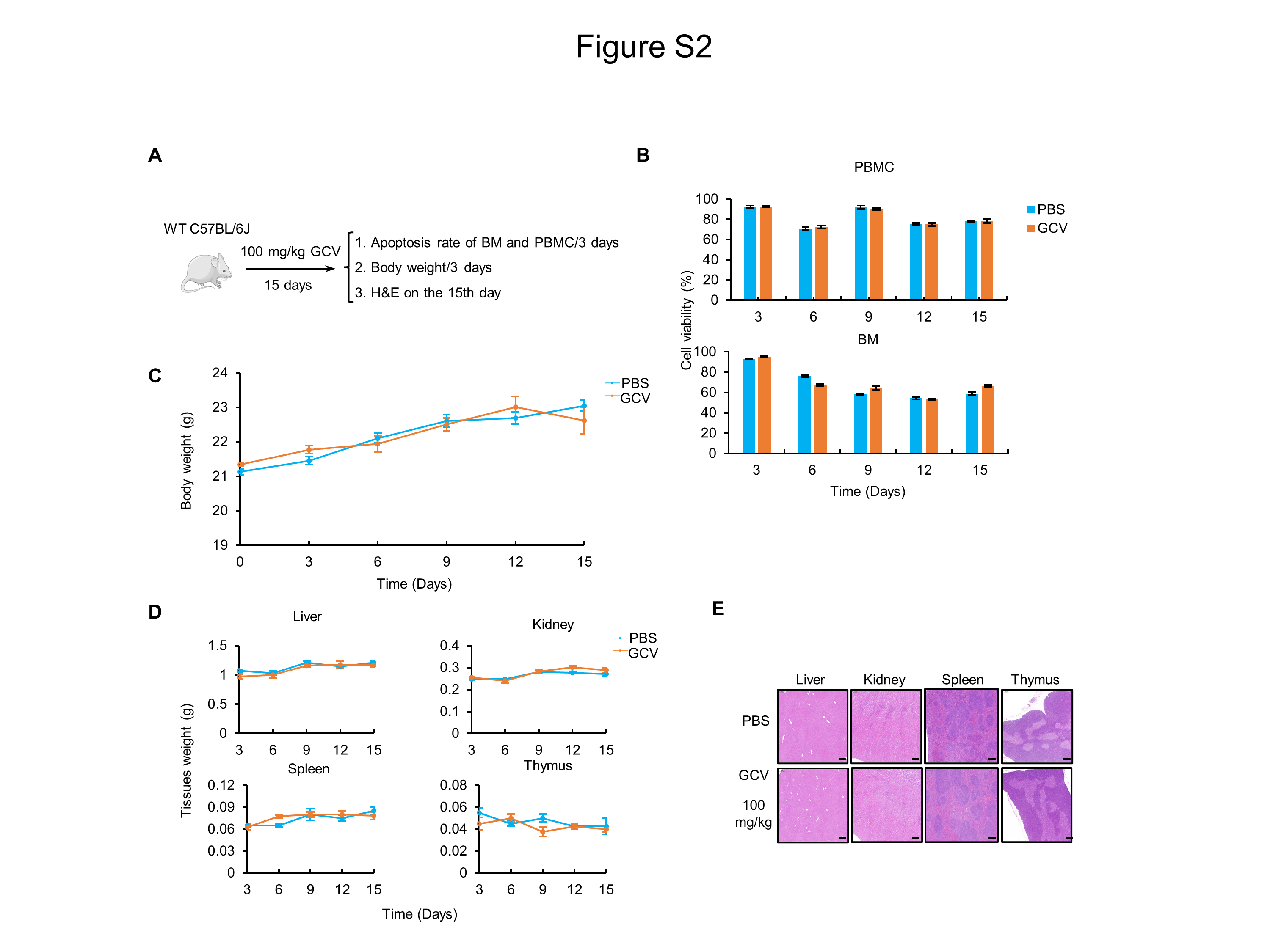

Supplement: Supplementary file 2 — Figure S2. Safety evaluation of the GCV treatment dose of 100 mg/kg. (A) Schematic illustration of the experimental design to test the toxicity of administering 100 mg/kg GCV for 15 days. WT C57BL/6J mice were treated with GCV (100 mg/kg) or PBS once a day for 15 days by peritoneal injection. The viability of BM cells and PBMCs was analysed on day 0, day 3, day 6, day 9, day 12, and day 15 by staining with annexin V and PI. The body weights were measured every 3 days. Tissue weights were measured on day 0, day 3, day 6, day 9, day 12, and day 15. H&E staining of tissue sections from each group of mice was performed on the 15th day. (B‐E) 15 days of continuous 100 mg/kg GCV treatment does not induce a significant decrease in cell viability (B), body weight (C), tissue weight (D), or tissue structure (E) compared with the control group. Scale bar: 200 μm. Data are shown as the mean values±SEM, n = 20; *, P < 0.05; **, P < 0.01; ***, P < 0.001; ****, P < 0.0001; ns, not significant. [file CPR-56-e13472-s003.tif]

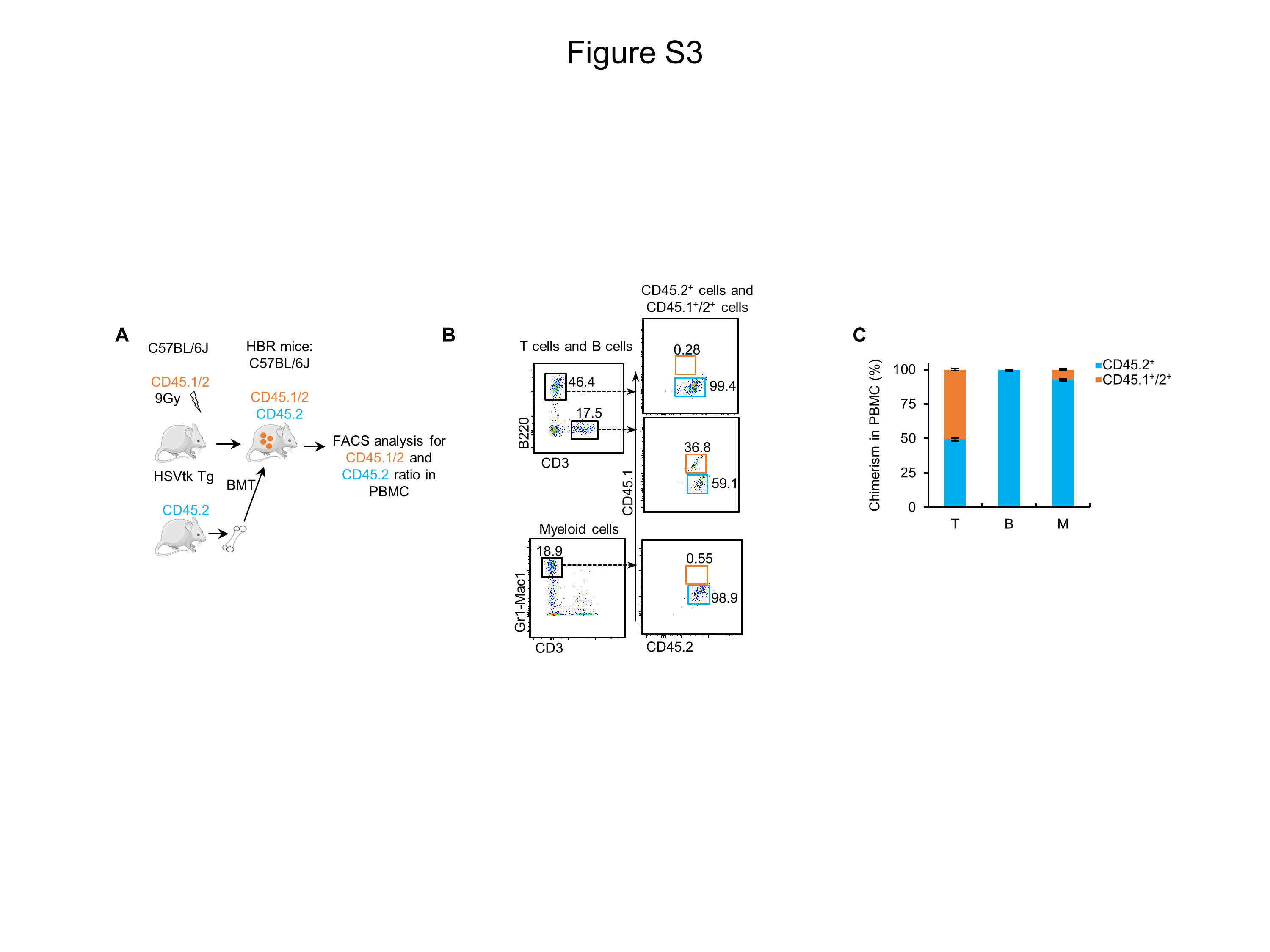

Supplement: Supplementary file 3 — Figure S3. HBR mice are generated by transplantation of bone marrow cells into HSVtk Tg mice. (A) Schematic of the procedure for constructing HBR mice. (B) Schematic overview of the FACS gating strategy. The left panels show the FACS sorting of PBMCs: B220+ B cells, Gr1‐Mac1+ myeloid cells, and CD3+ T cells. These populations are then gated for CD45.1+ cells, CD45.2+ cells, and CD45.1+/2+ cells (right panels). (C) The percentage of CD45.2+ cells and CD45.1+/2+ cells in HSVtk‐BM recipient (HBR) mice, determined by FACS as shown in B. T: T cells, marker is CD3+; B: B cells, marker is B220+; M: myeloid cells, marker is Gr1‐Mac1+. Data are shown as the mean values±SEM, n = 133. [file CPR-56-e13472-s005.tif]

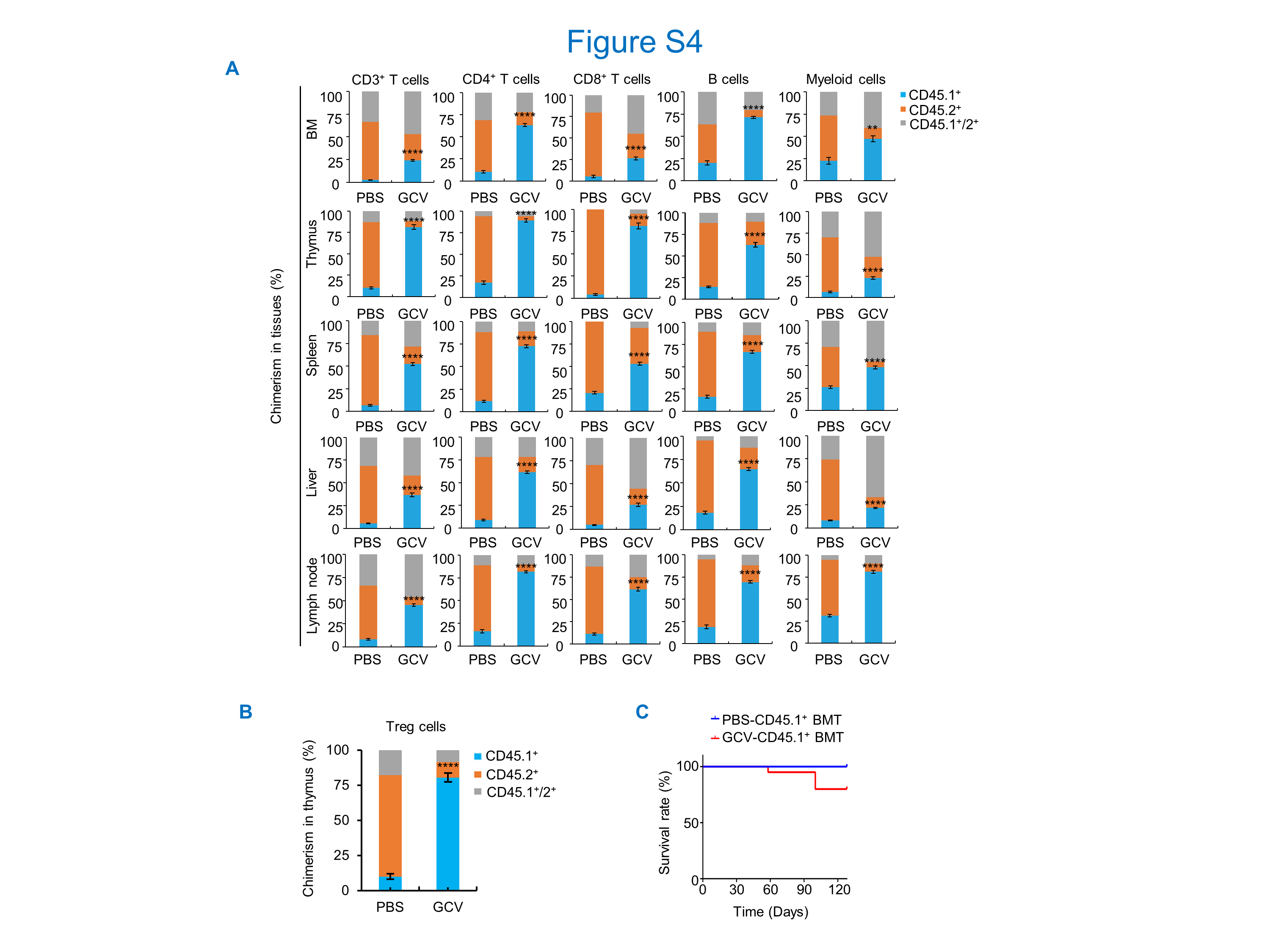

Supplement: Supplementary file 4 — Figure S4. Mice receiving syngeneic transplants exhibit a high level of donor cell chimerism and a high survival rate. (A) T cells (CD3+/CD4+/CD8+ cells), B cells (B220+ cells), and myeloid cells (Gr1‐Mac1+ cells) in different tissues from the recipient mice in the GCV and PBS groups at 16 weeks after the transplantation of bone marrow from C57BL/6J (CD45.1) donor mice. (B) Statistical analysis of the chimerism ratios (CD45.1+ cell %) of CD4+ CD25+ FOXP3+ Treg cells in the thymus tissues detected by FACS analysis at the 16th week after BMT. (C) Survival curves of the mice in GCV and PBS group after syngeneic BMT. (GCV, ninitial = 20, nsurviving at week 16 = 16; PBS, n = 5; data are shown as the mean values±SEM; **,P < 0.01; ****, P < 0.0001) [file CPR-56-e13472-s006.tif]

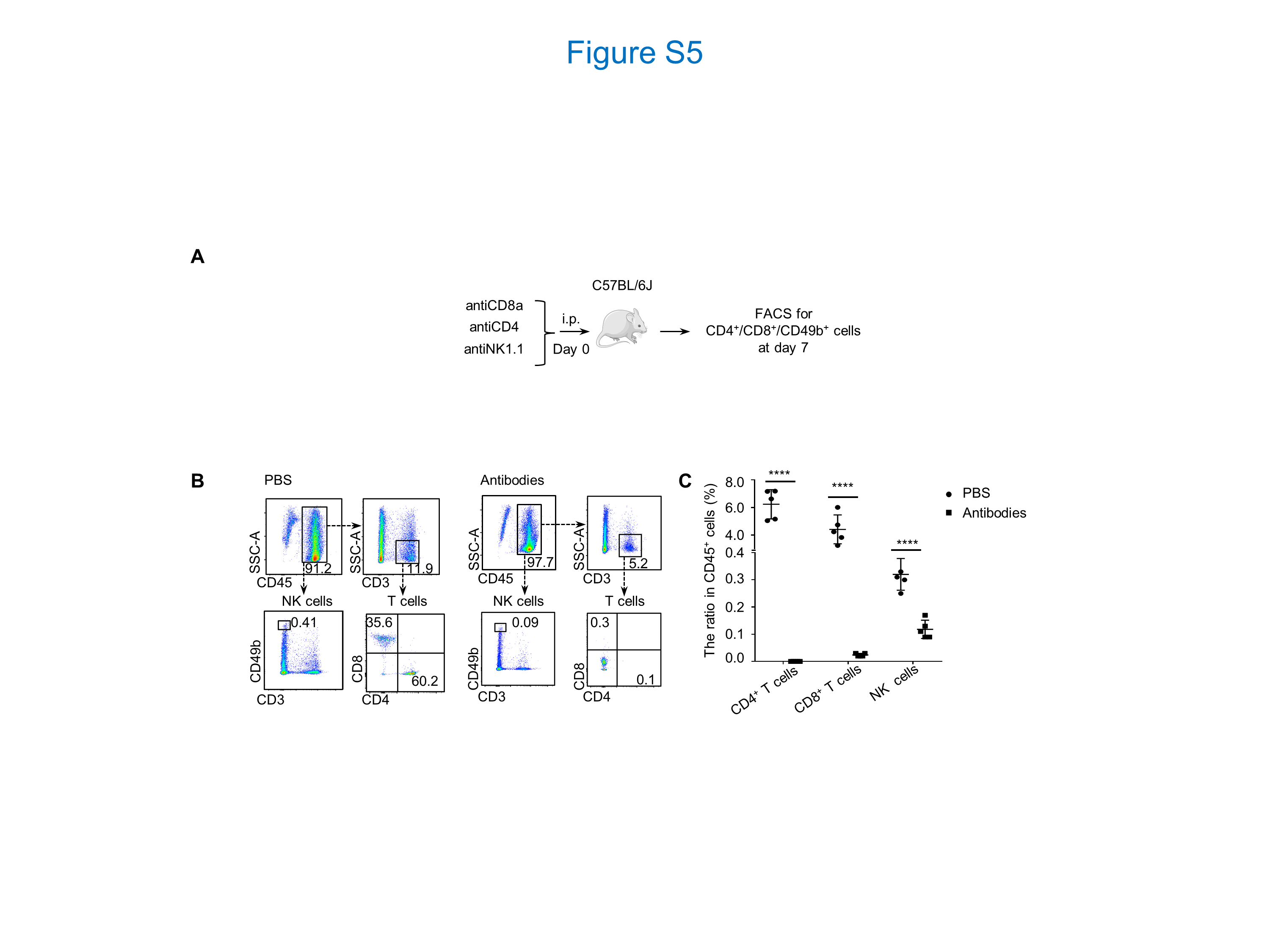

Supplement: Supplementary file 5 — Figure S5. CD4 + T cells, CD8 + T cells, and NK cells in the recipient mice are blocked by intraperitoneal injection of neutralising antibodies. (A) Schematic of the procedure for titrating the neutralising antibodies. WT C57BL/6J mice were intraperitoneally injected with neutralising antibodies (anti‐CD4: 200 μg; anti‐CD8: 200 μg; and anti‐NK1.1: 200 μg) or PBS at day 0. The percentage of CD4+ helper T cells, CD8+ cytotoxic T cells, and CD49b+ natural killer cells (NK cells) in the PBMCs were analysed on day 7 (control group: PBS; experimental group: Antibodies). (B) The distributions of CD4+ T cells, CD8+ T cells, and CD49b+ NK cells in the PBMCs from each group of mice were detected by FACS at day 7 after treatment. (C) The percentages of CD4+ T cells, CD8+ T cells, and NK cells are significantly lower in the Antibodies group than in the PBS group. (n = 5, mean values±SEM, ****P < 0.0001). [file CPR-56-e13472-s004.tif]

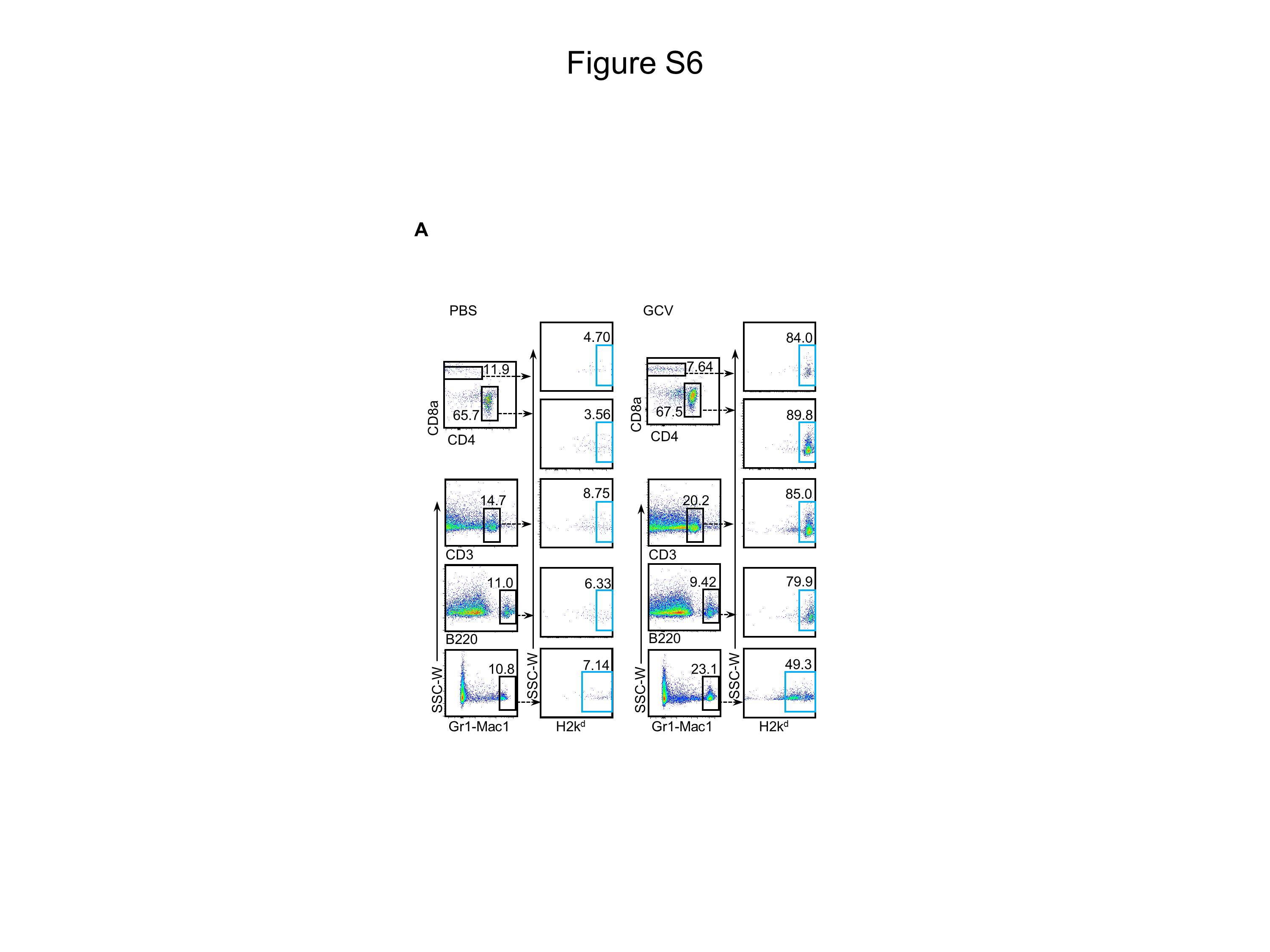

Supplement: Supplementary file 6 — Figure S6. Mice receiving allogeneic transplants exhibit a high level of donor cell chimerism in different tissues. (A) FACS analysis of the chimerism of T cells (CD3+/CD4+/CD8+ cells), B cells (B220+ cells), and myeloid cells (Gr1‐Mac1+ cells) in PBMCs from the recipient mice in the GCV and PBS groups at 16 weeks after transplantation of bone marrow (BM) cells from Balb/c (H2Kd) donor mice. (B) Representative FACS diagram of chimerism of LSK cells (Lineage− Sca1+ c‐Kit+) in BM of the GCV and PBS groups at 16 weeks after the transplantation of BM from Balb/c (H2Kd) donor mice. (C) FACS analyses of Treg cells (CD4+ CD25+ Foxp3+) in thymus tissues from the mice in the GCV and PBS groups at 16 weeks after receiving BM transplants from Balb/c (H2Kd) donors. (D) Chimerism levels of T cells (CD3+/CD4+/CD8+ cells), B cells (B220+ cells), and myeloid cells (Gr1‐Mac1+ cells) in different tissues from the mice in the GCV and PBS groups at 16 weeks after receiving BM transplants from Balb/c (H2Kd) donors. (E) Representative FACS diagram of the chimerism level of rat CD45 (rCD45) cells in PBMCs from each group of mice at 16 weeks after transplantation of BM or Lin− cells from F344 rats. (GCV, ninitial = 20, nsurviving at week 16 = 1; PBS, n = 5; data are shown as the mean values±SEM). [file CPR-56-e13472-s002.zip › CPR_13472_Figure S6-1.tif]

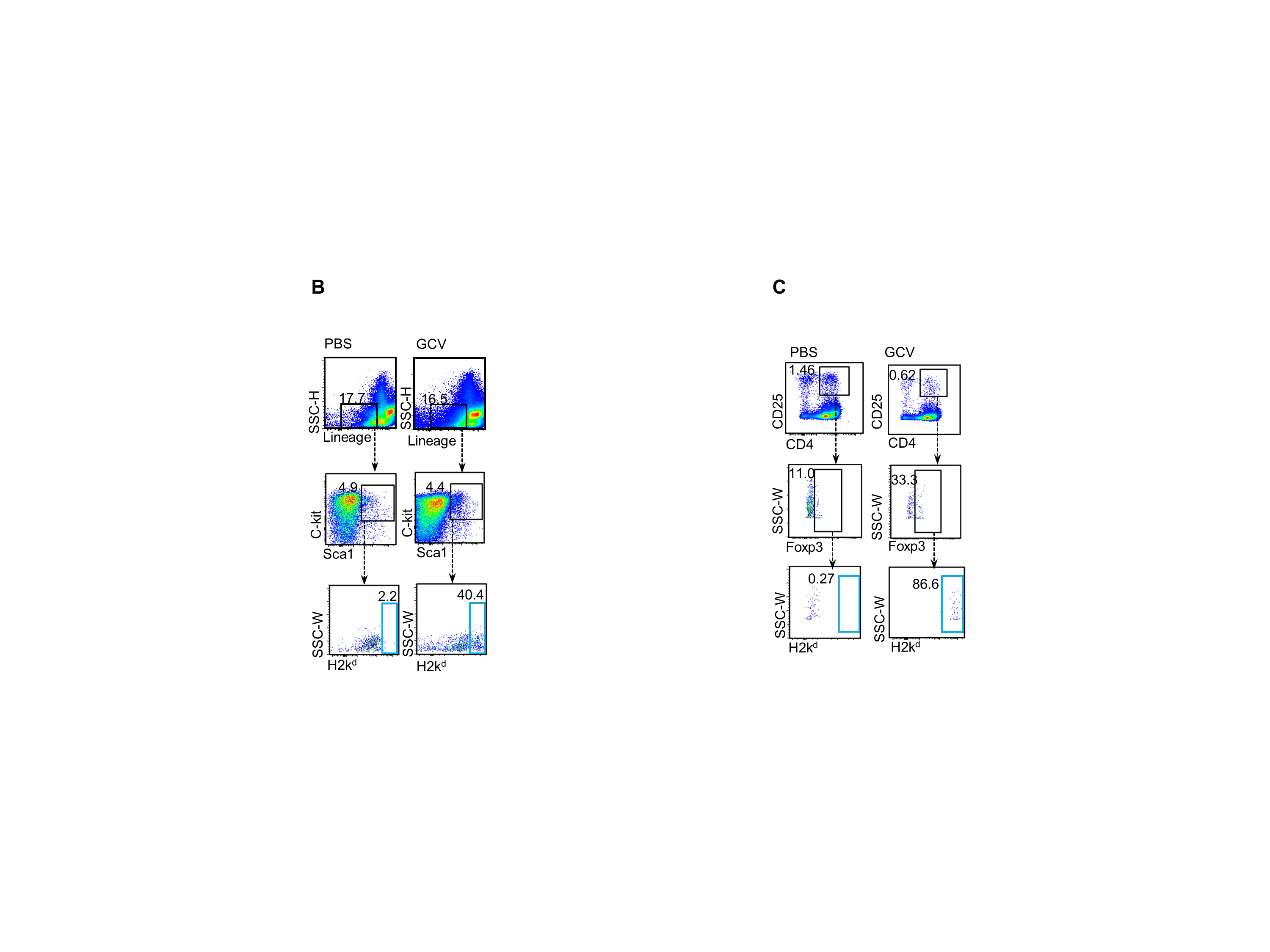

Supplement: Supplementary file 6 — Figure S6. Mice receiving allogeneic transplants exhibit a high level of donor cell chimerism in different tissues. (A) FACS analysis of the chimerism of T cells (CD3+/CD4+/CD8+ cells), B cells (B220+ cells), and myeloid cells (Gr1‐Mac1+ cells) in PBMCs from the recipient mice in the GCV and PBS groups at 16 weeks after transplantation of bone marrow (BM) cells from Balb/c (H2Kd) donor mice. (B) Representative FACS diagram of chimerism of LSK cells (Lineage− Sca1+ c‐Kit+) in BM of the GCV and PBS groups at 16 weeks after the transplantation of BM from Balb/c (H2Kd) donor mice. (C) FACS analyses of Treg cells (CD4+ CD25+ Foxp3+) in thymus tissues from the mice in the GCV and PBS groups at 16 weeks after receiving BM transplants from Balb/c (H2Kd) donors. (D) Chimerism levels of T cells (CD3+/CD4+/CD8+ cells), B cells (B220+ cells), and myeloid cells (Gr1‐Mac1+ cells) in different tissues from the mice in the GCV and PBS groups at 16 weeks after receiving BM transplants from Balb/c (H2Kd) donors. (E) Representative FACS diagram of the chimerism level of rat CD45 (rCD45) cells in PBMCs from each group of mice at 16 weeks after transplantation of BM or Lin− cells from F344 rats. (GCV, ninitial = 20, nsurviving at week 16 = 1; PBS, n = 5; data are shown as the mean values±SEM). [file CPR-56-e13472-s002.zip › CPR_13472_Figure S6-2.tif]

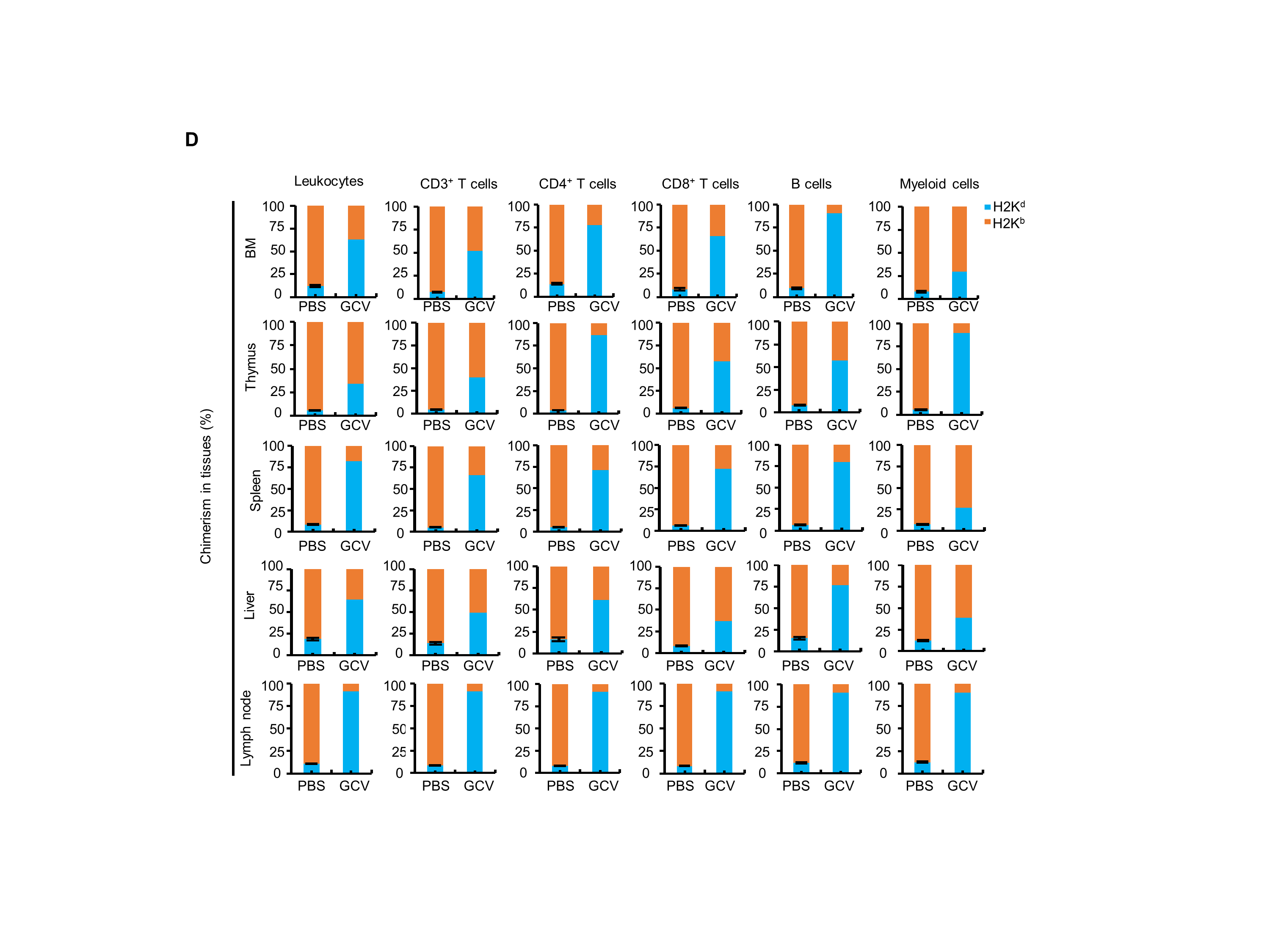

Supplement: Supplementary file 6 — Figure S6. Mice receiving allogeneic transplants exhibit a high level of donor cell chimerism in different tissues. (A) FACS analysis of the chimerism of T cells (CD3+/CD4+/CD8+ cells), B cells (B220+ cells), and myeloid cells (Gr1‐Mac1+ cells) in PBMCs from the recipient mice in the GCV and PBS groups at 16 weeks after transplantation of bone marrow (BM) cells from Balb/c (H2Kd) donor mice. (B) Representative FACS diagram of chimerism of LSK cells (Lineage− Sca1+ c‐Kit+) in BM of the GCV and PBS groups at 16 weeks after the transplantation of BM from Balb/c (H2Kd) donor mice. (C) FACS analyses of Treg cells (CD4+ CD25+ Foxp3+) in thymus tissues from the mice in the GCV and PBS groups at 16 weeks after receiving BM transplants from Balb/c (H2Kd) donors. (D) Chimerism levels of T cells (CD3+/CD4+/CD8+ cells), B cells (B220+ cells), and myeloid cells (Gr1‐Mac1+ cells) in different tissues from the mice in the GCV and PBS groups at 16 weeks after receiving BM transplants from Balb/c (H2Kd) donors. (E) Representative FACS diagram of the chimerism level of rat CD45 (rCD45) cells in PBMCs from each group of mice at 16 weeks after transplantation of BM or Lin− cells from F344 rats. (GCV, ninitial = 20, nsurviving at week 16 = 1; PBS, n = 5; data are shown as the mean values±SEM). [file CPR-56-e13472-s002.zip › CPR_13472_Figure S6-3.tif]

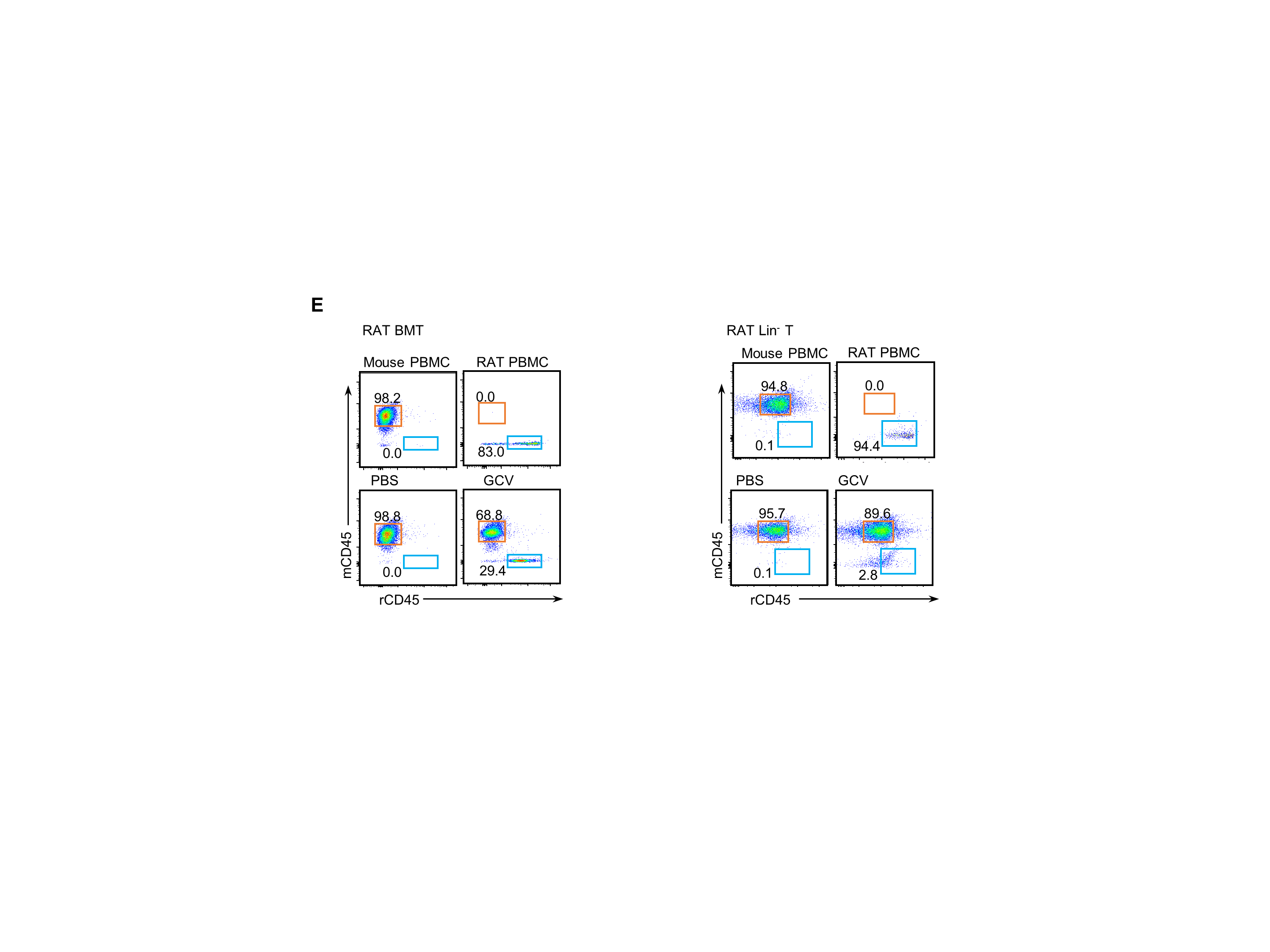

Supplement: Supplementary file 6 — Figure S6. Mice receiving allogeneic transplants exhibit a high level of donor cell chimerism in different tissues. (A) FACS analysis of the chimerism of T cells (CD3+/CD4+/CD8+ cells), B cells (B220+ cells), and myeloid cells (Gr1‐Mac1+ cells) in PBMCs from the recipient mice in the GCV and PBS groups at 16 weeks after transplantation of bone marrow (BM) cells from Balb/c (H2Kd) donor mice. (B) Representative FACS diagram of chimerism of LSK cells (Lineage− Sca1+ c‐Kit+) in BM of the GCV and PBS groups at 16 weeks after the transplantation of BM from Balb/c (H2Kd) donor mice. (C) FACS analyses of Treg cells (CD4+ CD25+ Foxp3+) in thymus tissues from the mice in the GCV and PBS groups at 16 weeks after receiving BM transplants from Balb/c (H2Kd) donors. (D) Chimerism levels of T cells (CD3+/CD4+/CD8+ cells), B cells (B220+ cells), and myeloid cells (Gr1‐Mac1+ cells) in different tissues from the mice in the GCV and PBS groups at 16 weeks after receiving BM transplants from Balb/c (H2Kd) donors. (E) Representative FACS diagram of the chimerism level of rat CD45 (rCD45) cells in PBMCs from each group of mice at 16 weeks after transplantation of BM or Lin− cells from F344 rats. (GCV, ninitial = 20, nsurviving at week 16 = 1; PBS, n = 5; data are shown as the mean values±SEM). [file CPR-56-e13472-s002.zip › CPR_13472_Figure S6-4.tif]
